# Rare Earth Elements Removal from Water Using Natural Polymers

**DOI:** 10.1038/s41598-017-18623-0

**Published:** 2018-01-10

**Authors:** Adina Negrea, Andreea Gabor, Corneliu Mircea Davidescu, Mihaela Ciopec, Petru Negrea, Narcis Duteanu, Alina Barbulescu

**Affiliations:** 1 Politehnica University Timişoara, Faculty of Industrial Chemistry and Environmental Engineering, Victoriei Square Nr. 2, 300006 Timişoara, Romania; 2 Ovidius University of Costanta, Romania,124, Mamaia Blvd., 900527 Constanta, Romania

## Abstract

Adsorption of rare earth metals, Eu (III) and Nd (III) was investigated on a new environmental friendly material, thiourea functionalized cellulose. Before usage, the synthesized material was characterized by Fourrier Transform Infrared spectroscopy and energy dispersive X-ray analysis. The influence of adsorption parameters (adsorbent dosage, time, temperature and initial metal concentration) on adsorption capacity was investigated. Experimental data were fitted by using the pseudo-first-order and pseudo-second-order kinetic models. Simultaneously thermodynamic and equilibrium studies have been carried out using Langmuir, Freundlich and Sips isotherm. Maximum adsorption capacities were reached in 30 minutes at 298 K having the value of 27 mg/g for Eu (III) and 73 mg/g for Nd (III).

## Introduction

Rapid industrial development during last decades involves development and extensive usage of special materials in different commercial products^1–3^. The usage of heavy metals in industry introduces a large amount of toxic metals into atmosphere as well as into the aquatic and terrestrial environment^4^. Physical and chemical properties of the rare earth elements (REEs), correlated with their electronic, optical, and metallurgical properties make them perfect candidates for specific applications in cutting-edge technologies^5–7^. Wide spread of rare earth elements is due to their applications in many fields as special alloys, magnets, catalysts, and to their usage into equipments as batteries, sensors, energy efficient lighting, electric vehicles^7–9^, nuclear technologies, telecommunications, security systems^1–3^, medical application, and as active components of some fertilizers^10^. Industrial development is leading at real increase of consumption that can lead at some technical issues of waste disposal, especially due to the heavy metals content. Inadequate waste disposal leads at higher increase of public exposure at toxic compounds having negative effects on environment and human health^11^. It was proved the REE’s bioaccumulation through the food chain can cause ailments due to the exposure of humans at low concentration of REEs^6,10^.

A viable alternative is the proper treatment of electrical and electronic compounds which have reached their lifetime, and that can become an alternative resource for many metals including REEs^8^. Applications from high technology field require high purity of REEs, with the content of non-rare earth impurity strictly limited, due to the negative influence of the impurities on the properties of desired rare earth material^5^. Nowadays, the extensive usage of REEs leads to an increase of environmental pollution.

Last decades, several methods (chemical precipitation, ion exchange extraction, coagulation, flocculation, liquid – liquid extraction, solid – phase extraction, biosorption, classical adsorption on different adsorbents) have been developed and used for metal removal from wastewaters^7,12^. The most effective and attractive one is represented by the adsorption, due to the advantages associated with the inexistence of chemical sludge and its highly removal efficiency^12^.

Taking into account the environmental protection, new classes of economic and environmental friendly adsorbents must be developed and produced. Biopolymers represent a possible new class of adsorbents. Polysaccharides are widely available, biological polymers presenting a remarkable structural diversity. Based on their properties, they became an indispensable material for medicine, pharmaceutical industry, food, textile industry and also for environmental protection^13,14^. One of the simplest (biopolymer) polysaccharide is cellulose, which is a linear polysaccharide presenting no branches or substituents^15^ and is often considered as the most abundant polymer produced in nature^13,15^. Being a natural product, represents a renewable biopolymer and can be considered as a promising environmental friendly adsorbent^16^. Compared with other bi-products, cellulose presents a bigger number of hydroxyl groups which can be easily modified, improving this way the synthesis yield and increasing his commercial value by making it eligible for new technological applications^16,17^.

In this study, cellulose was functionalized by impregnation with thiourea in order to get a new environmental friendly adsorbent material and was used for the adsorption of Nd (III) and Eu (III) ions from aqueous solutions. By performing kinetic, equilibrium and thermodynamic studies, we proved that beside a proper solid support, usage of thiourea as extractant has improved adsorption properties of cellulose.

## Methods

### Chemicals and characterization

All used chemicals are analytical grade and were purchased for Sigma- Aldrich. Functionalized adsorbent material was characterized by using FTIR and also EDX, in order to prove that the used technique leads at functionalized cellulose. After all adsorption experiment concentration of RREs into the effluent was measured using ICP- MS.

ICP-MS represent the most powerful analytical technique for detecting REEs, having the capability of multi-element detection over a wide concentration range, achieving extremely low detection limits and low mass interference^6^.

### Functionalization of the solid support

In preliminary attempt was studied the influence of cellulose thiourea ratio on to the maximum adsorption capacity, so same amount of cellulose (1 gram) was mixed with three different solutions containing different amount of adsorbent (5 mL solution containing 0.05, 0.1 and respectively 0.2 g of thiourea dissolved in pure ethanol). Cellulose samples were maintained in contact with adsorbent solution for 24 hours, and after that was filtered, washed, and dried for minimum 24 hours at a suitable temperature. Obtained modified cellulose was used in adsorption experiment in order to find how the ratio cellulose: thiourea is influencing the adsorption of europium and neodymium ions. So in this stage synthesized material was kept in contact for 60 minutes with solutions containing 50 mg L^−1^ Eu and Nd ions, after that the solution was filtered and were determined the residual concentrations of Eu and Nd ions after adsorption, obtaining in this way the maximum adsorption capacity (results presented in Table 1). Based on depicted data can observe that the maximum adsorption capacity have a constant value, so we choose for further experiment the ratio cellulose: thiourea 1:0.1.

**Table 1:** Preliminary attempts for determination of optimum ratio cellulose: thiourea.

Eu				Nd			
Ci = 50 mg/L				Ci = 50 mg/L			
ratio cellulose:thiourea	1:0.05	1:0.1	1:0.2	ratio cellulose:thiourea	0.05:1	0.1:1	0.2:1
Q	10.4	10.3	10.6	q	11.6	11.6	11.7

(Ci – initial concentration, q – adsorption capacaity).

Taking in account the results obtained during preliminary attempts cellulose was functionalized by mixing 5 g of cellulose with 25 mL solution containing 0.5 g of thiourea dissolved in 25 mL ethanol. Cellulose was maintained in contact with the adsorbent for 24 hours, and then the modified cellulose was filtered, washed, and dried for 24 hours at 323 K. The new prepared material was characterized using two different methods: Fourrier Transform Infrared Spectroscopy (FTIR) and Energy Dispersive X-Ray Analysis (EDX). FTIR spectra was recorded on Shimadzu Presige-21 FTIR spectrophotometer in the range 4000–400 cm^−1^ by using KBr pellets technique, and the EDX spectra was recorded using Quanta FEG 250 scanning electron microscopy.

### Adsorption experiments

Adsorption experiments were carried out to define the influence of important parameters: adsorbent dosage, contact time, temperature and initial concentration of metal ions onto the adsorption efficiency.

All Eu (III) and Nd (III) solutions used during adsorption experiments were prepared from stock solutions with 1 g L^−1^ concentration, through proper dilution.

Adsorbent dosage influence was studied by using different amounts of functionalized cellulose (0.05, 0.1, 0.2, 0.3, 0.4, 0.5 g) which were mixed one hour with 25 mL solution containing 50 mg L^−1^ of metal ions.

Contact time influence was studied using four samples 0.1 g of functionalized material mixed with 25 mL solutions containing 50 mg metallic ions per liter, and kept in contact for 15, 30, 45 and 60 minutes.

In order to investigate the temperature influence, the same amount of metallic solution, with the same concentration was mixed with 0.1 g of functionalized adsorbent and kept at 298, 308, and 318 K for 30 minutes.

The influence of the initial metal concentration was followed up by mixing for 30 minutes at 298 K, samples of 0.1 g functionalized material with 25 mL solution containing 10, 50, 100, 150, 200, 250, 300 mg of ionic metals per liter, for both studied metallic ions.

All samples were mixed in a Julabo SW23 mechanical shaker bath at 200 rotation min^−1^, filtered after and the metallic ions residual concentration was analyzed by inductively coupled plasma mass spectrometer – ICPMS Bruker Aurora M90.

The stages of our research are the following:

1. Characterization of the functional material, by using the Fourrier transformed infrared spectroscopy (FTIR) and the electron dispersive X-Ray analysis (EDX)
2. Study of the influence of adsorbent dosage on the adsorption of Eu (III) and Nd (III)
3. The kinetic studies and activation energy
  In order to determine adsorption process kinetics for the studied material, experimental data were modeled using two different kinetic models: Lagergren pseudo-first -order model and Ho & McKay pseudo-second -order model.
  The pseudo-first -order model^18^ is described by the equation (1):
  1 $$\mathrm{ln}({q}_{e}-{q}_{t})=\,\mathrm{ln}\,{q}_{e}-{k}_{1}t$$
  where *t* is the contact time (min). *q*_e_ is the adsorption capacity at equilibrium (mg/g), *q*_*t*_ is the adsorption capacity at the time *t*, and *k*_1_ is the adsorption constant rate (1/min).
  The pseudo-second -order model^19^ can be described by equation (2)
  2 $$\frac{t}{{q}_{t}}=\frac{1}{{k}_{2}{q}_{e}^{2}}+\frac{t}{{q}_{e}}$$
  where *t* is the contact time (min), *q*_e_ is the adsorption capacity at equilibrium (mg/g), *q*_t_ is the adsorption capacity at time *t* and *k*_2_ the adsorption rate constant (g/mg∙min).
  The most important role in adsorption processes is played by the activation energy, which shows if the studied adsorption is a chemical or a physical process^20–27^. This energy can be evaluated by using the Arrhenius equation (equation (3)) using the adsorption rate constant, computed from the pseudo-second-order kinetic model:
  3 $$\mathrm{ln}\,{k}_{2}=\,\mathrm{ln}\,A-\frac{E}{RT}$$
  where *k*_2_ is the pseudo-second-order rate constant (g/min∙mg), *A* is the Arrhenius constant (min∙g/mg), *E* is the activation energy (kJ/mol), *R* the ideal gas constant (8.314 J/mol·K), and *T* represent absolute temperature (K).
4. Thermodynamic studies
  Thermodynamic studies were performed in order to establish if the studied adsorption processes are spontaneous or not. Therefore, it was necessary to determine the value of the free Gibbs energy for studied adsorption processes.
  Gibbs free energy can be computed from the Gibbs-Helmholtz relation (equation 4):
  4 $${\rm{\Delta }}{G}^{{\rm{o}}}={\rm{\Delta }}{H}^{{\rm{o}}}-T{\rm{\Delta }}{S}^{{\rm{o}}}$$
  where Δ*S*° is the standard entropy change and Δ*H*° is the standard enthalpy change.
  Standard entropy and enthalpy changes can be evaluated from van’t Hoff equation (equation 5):
  5 $$\mathrm{ln}\,{K}_{{\rm{d}}}=\frac{{\rm{\Delta }}{S}^{{\rm{o}}}}{R}-\frac{{\rm{\Delta }}{H}^{{\rm{o}}}}{RT},$$
  where *K*_d_ is the equilibrium constant, *T* is the absolute temperature (K), *R* the ideal gas constant (8.314 J/mol∙K).
  The equilibrium constant *K*_d_ is given by the equation (6)
  6 $${K}_{{\rm{d}}}=\frac{{q}_{{\rm{e}}}}{{C}_{{\rm{e}}}},$$
  *q*_e_ being the equilibrium adsorption capacity (mg/g), and *C*_e_ the equilibrium concentration (mg/L).
5. Equilibrium studies
  Firstly, the adsorption isotherms have been determined in order to elucidate the adsorption mechanism^19,28^. Secondly, the experimental data were fitted by using three different non-linear isotherm models: Langmuir, Freundlich and Sips.
  Langmuir’s model^29^ assumes that in all cases adsorption is accomplished by coating homogenous adsorbent surface with a monolayer of adsorbate, because in this case all active sites over the adsorbent surface are identical, and in same time all adsorbed molecules have the same activation energy. The non-linear Langmuir isotherm is described by equation (7):
  7 $${q}_{{\rm{e}}}=\frac{{q}_{{\rm{L}}}{K}_{{\rm{L}}}{C}_{{\rm{e}}}}{1+{K}_{{\rm{L}}}{C}_{{\rm{e}}}}.$$
  where: *q*_e_ is the equilibrium adsorption capacity (mg/g), *C*_e_ is the equilibrium concentration of adsorbent in the solution (mg/L), *q*_L_ (mg/g) represents the maximum adsorption capacity, and *K*_L_ is the Langmuir constant connected to the free energy of adsorption.
  Freundlich isotherm model^30^ assumes that the adsorption can take place also on heterogeneous surfaces as a multilayer adsorption. The non-linear Freundlich adsorption model is described by equation (8):
  8 $${q}_{{\rm{e}}}={K}_{{\rm{F}}}{C}_{{\rm{e}}}^{1/{n}_{{\rm{F}}}}.$$
  where: *q*_e_ is the equilibrium adsorption capacity (mg/g), *C*_e_ is the equilibrium concentration of the adsorbent in solution (mg/L), *K*_F_ and *n*_F_ are specific constants that are connected to the relative adsorption capacity of the adsorbent material and the adsorption intensity.
  Sips’ adsorption model^31^ represents a combination of the Langmuir and Freundlich models: at low concentrations, the adsorption processes have Freundlich properties, and at higher concentrations have Langmuir properties. Sips’ adsorption model is described by equation (9):
  9 $${q}_{e}=\frac{{q}_{s}{K}_{s}{C}_{e}^{1/{n}_{s}}}{1+{K}_{s}{C}_{e}^{1/{n}_{s}}}.$$
  where: *q*_s_ is the maximum adsorption capacity (mg/g), *K*_s_ is a constant related to the adsorption capacity of the material (mg/g) and *n*_s_ is the heterogeneity factor.
  Adsorption isotherms are used in order to better understand the adsorption process and in order to elucidate the adsorption mechanism.
6. Mathematical modeling of the evolution of q – adsorption capacity function of the REEs concentration at 298 K and of q function of time and temperature was performed. The first model is of Weibull type and the second one, linear multiple model.

## Results and Discussions

### Characterization of the functionalized material

#### FTIR characterization

**Figure 1:**
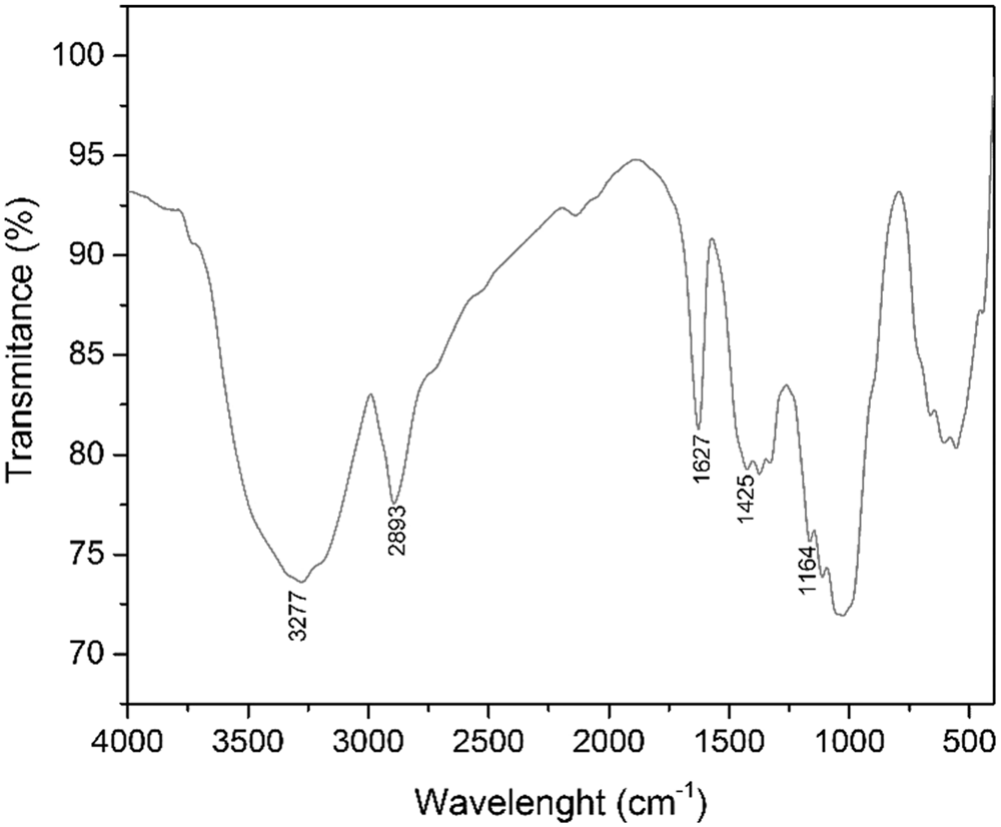
FTIR spectra of cellulose functionalized with thiourea.

In order to identify if was obtained the functionalized cellulose was compared the FTIR spectra of functionalized cellulose with FTIR spectra of pure cellulose and pure thiourea (depicted in Fig. 1). From spectra of pure compounds were identified characteristic bands of cellulose and thiourea. Into the functionalized cellulose spectra is expected that the thiourea specific bands have less intensity in comparison with the pure compound spectra.

In wavelength interval between 3500 and 3000 cm^−1^ can observe the large band located at 3277 cm^−1^ presenting a small plateau at 3160 cm^−1^ corresponding to the vibration of OH bonds. This large cellulose band include the asymmetric vibration of NH_2_ thiourea group located at 3395 cm^−1^ and the symmetric vibration of NH thiourea group located at 3179 cm^−1^. Band located at 2893 cm^−1^ is associated with the vibrations of cellulose glucopyranose cycle C-H bonds.

Intense band located at 1627 cm^−1^ into the FTIR spectra of functionalized cellulose is due to the elongation vibration of NH_2_ group, and is not a band associated with cellulose because in case of pure cellulose is observed a weak band at 1427.3 cm^−1^. Intense bands observed at 1464 cm^−1^ (vibration of CN bond + elongation of NH bond) and at 1395 cm^−1^ (vibration of C=S bond) in spectra of pure thiourea, appears also into the spectra of functionalized cellulose as bands with medium intensity at 1425, 1380 and 1290 cm^−1^. Bands observed at 1164 and 1100 cm^−1^ into the spectra of functionalized cellulose appears also into the spectra of pure cellulose at 1158 and 1104 cm^−1^ associated with vibrations of C-O and O-C-O cellulosic bonds.

Based on presented data can conclude that analyzed material is represented by the thiourea functionalized cellulose.

#### EDX analysis

**Figure 2:**
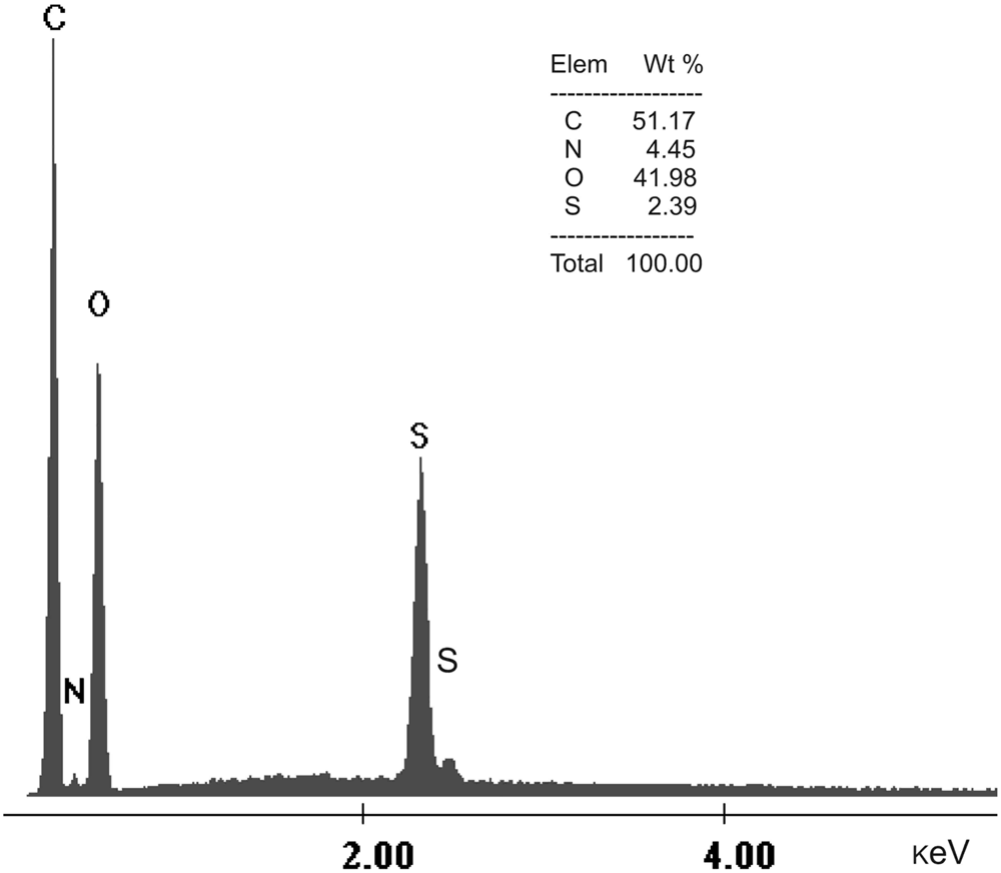
EDX spectra of cellulose functionalized with thiourea.

According to the EDX spectrum of cellulose functionalized with thiourea presented in Fig. 2, specific peaks belonging to elements from thiourea are visible (S peaks and N peak). These show a successfully functionalization of the natural polymer with thiourea extractant.

#### BET analysis

Cellulose and modified cellulose specific surface area were determined using a Micromeritics Brunauer-Emmett-Teller (BET) ASAP 2020 system. From BET adsorption isotherm (picture not showed) recorded for cellulose and functionalized cellulose were evaluated the specific surfaces areas. For pure cellulose was obtained a specific surface of 0.858 m^2^ g^−1^ and in case of functionalized cellulose was obtained a specific surface of 0.744 m^2^ g^−1^. From these data can observe a small decrease into the specific surface of the functionalized cellulose due to the thiourea functionalization.

### Eu (III) and Nd (III) adsorption onto cellulose functionalized with thiourea

In preliminary attempts were effectuated comparative tests in order to see how the functionalization is affecting the maximum adsorption capacity of cellulose. So, by using Eu and Nd solutions with initial concentrations of 50 mg L^−1^ were obtained the maximum adsorption capacities of 1.69 mg Eu ions per g of cellulose and 2.39 mg of Nd per g of cellulose in comparison with the maximum adsorption capacities of 10.3 and 11.6 obtained when the functionalized cellulose was used as adsorbent material.

### Influence of the adsorbent dosage

**Figure 3:**
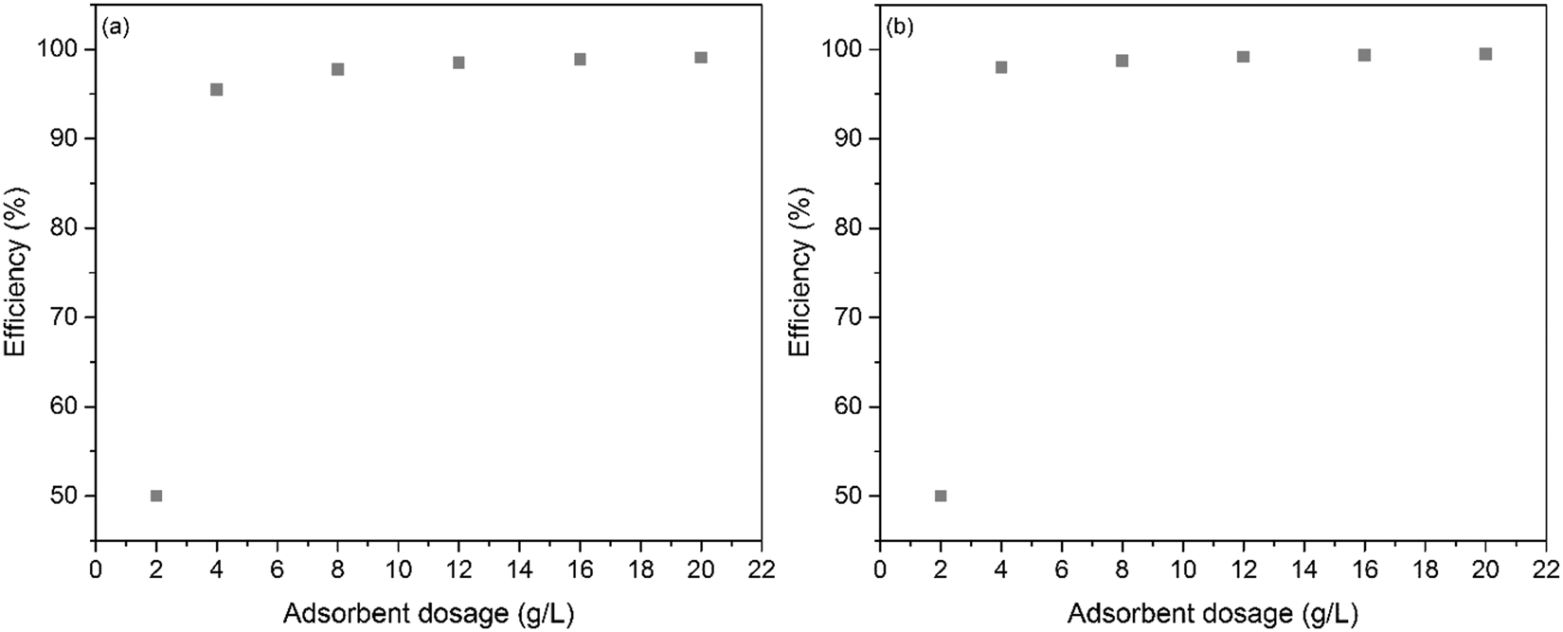
Adsorbent dosage influence on the adsorption of (**a**) Eu (III) and (**b**) Nd (III) (normalized at litter).

The influence of the adsorbent dosage on the adsorption of Eu (III) and Nd (III) on thiourea functionalized cellulose is depicted in Fig. 3.

It can be observed that when the used adsorbent was less than 4 g L^−1^, the adsorption efficiency for both metals was around 50%. By increasing the adsorbent quantity, the removal efficiency increases and remains approximately constant at 95%. Based on presented data, it can be concluded that the optimum amount of the adsorbent for maximum adsorption efficiency is 4 g L^−1^, amount used in all next experiments.

### Kinetic studies and activation energy

**Figure 4:**
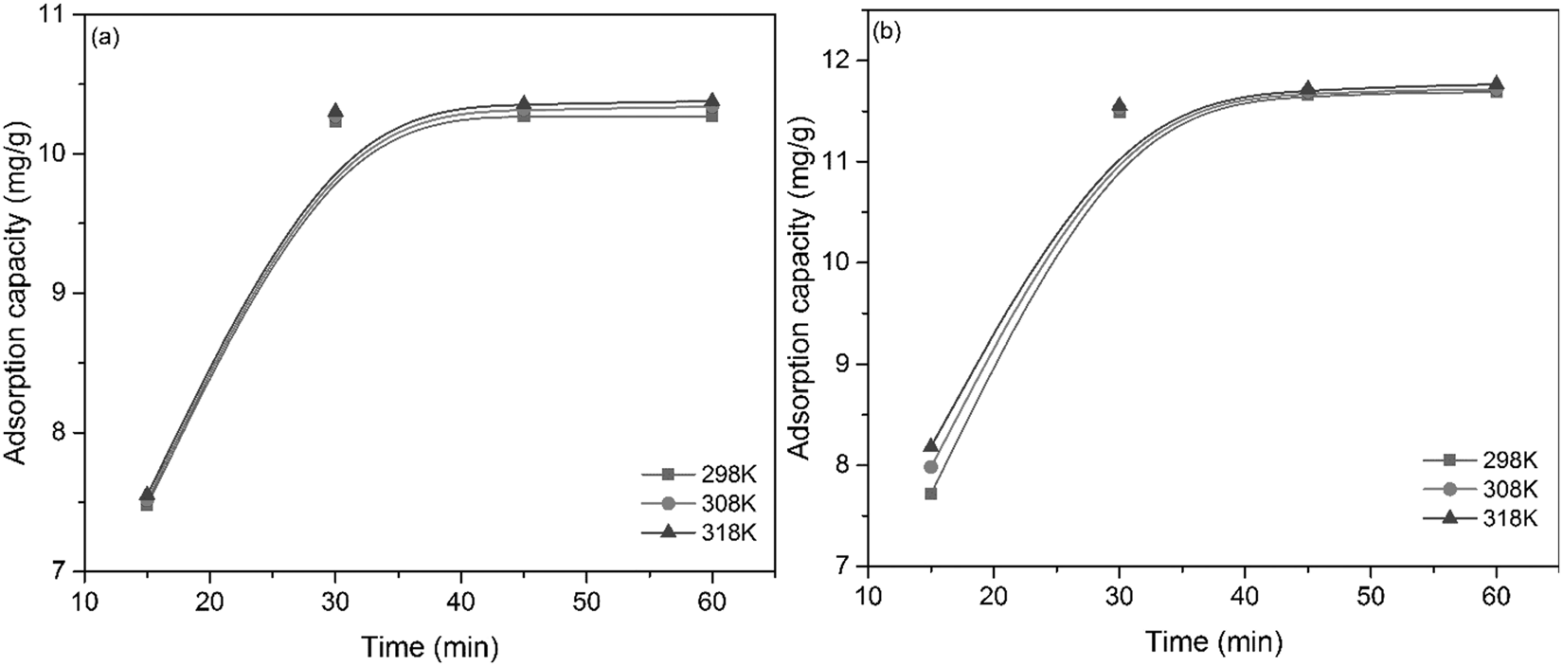
Effect of the contact time on the adsorption of (**a**) Eu (III) and (**b**) Nd (III) on the functionalized cellulose.

As expected, the contact time between the adsorbent and the contaminated solution have an important influence on the adsorption efficiency for Eu (III) and Nd (III) ions. The temperature influences the maximum adsorption capacity of the modified cellulose as well. Based on the data presented in Fig. 4, it can be remarked the increase of the adsorption capacity for both ions when the temperature rises from 298 to 318 K. The Eu and Nd ions adsorption efficiencies are reaching the maximum values (10.3 mg Eu (III) for each gram of adsorbent and 11.6 mg Nd (III) for each gram of adsorbent used) after 30 minutes at 298 K. Further increase of the contact time does not lead to the further augmentation of the adsorption efficiencies.

**Figure 5:**
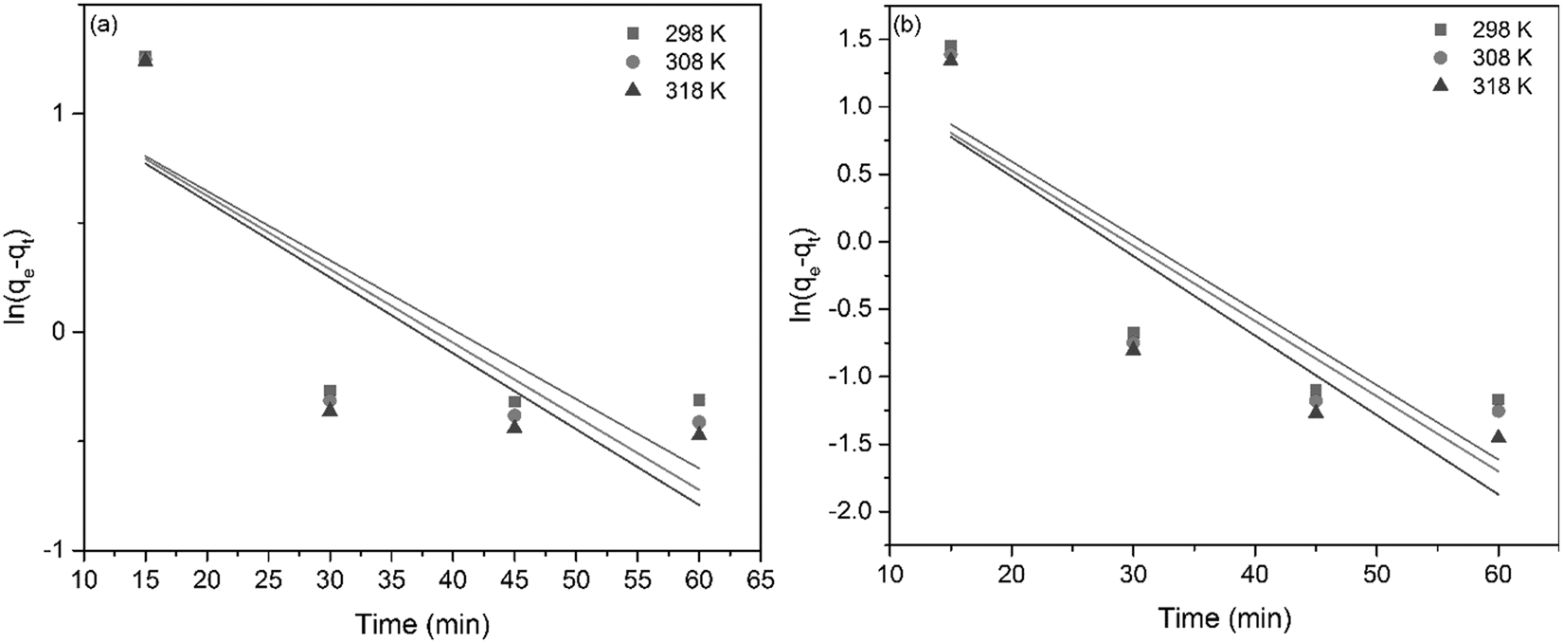
Pseudo-first-order kinetic plots of the adsorption of (**a**) Eu (III) and (**b**) Nd (III).

Figure 5 depict the plot of ln(*q*_*e*_ − *q*_*t*_) against *t* obtained by modeling the experimental data using Lagergren pseudo-first-order model. Adsorption rate constant *k*_1_ and correlation coefficient are calculated from lines slopes and from the intercept points.

**Figure 6:**
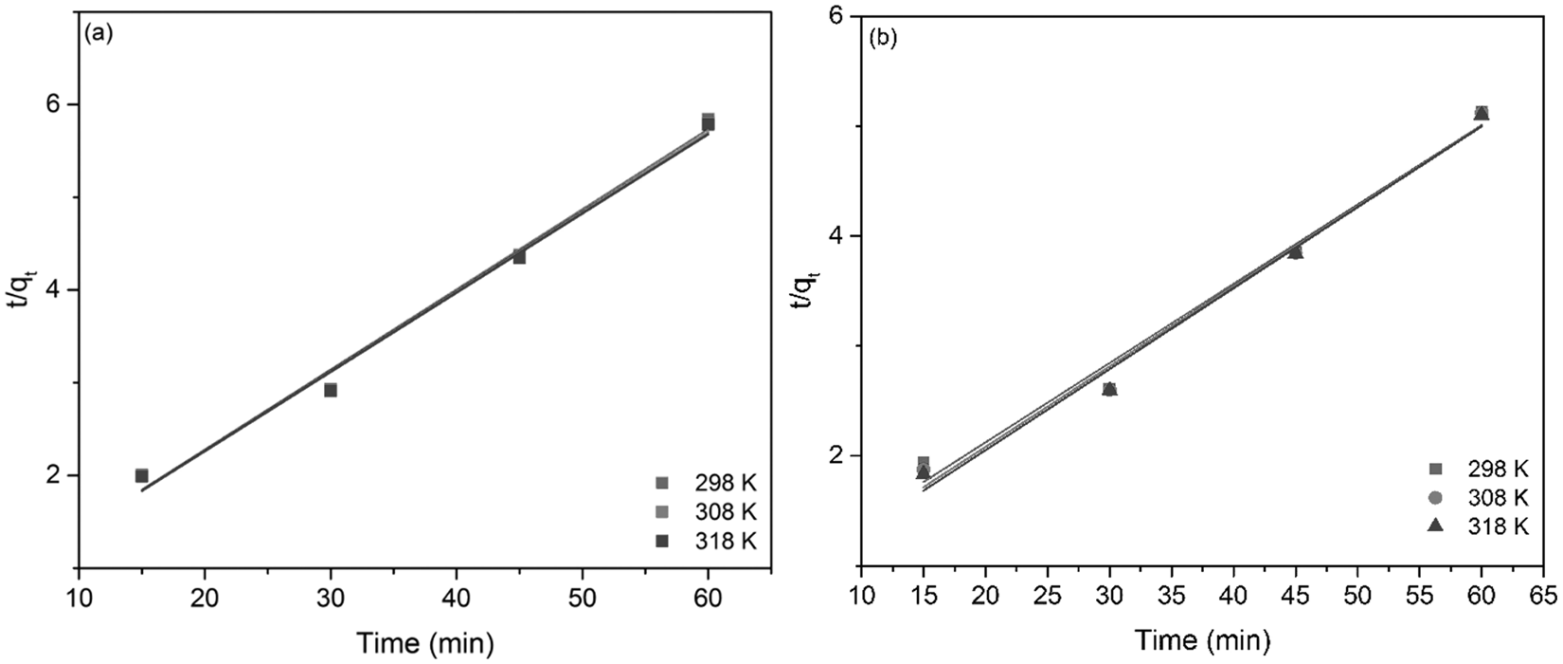
Pseudo-second-order kinetic plots of the adsorption of (**a**) Eu (III) and (**b**) Nd (III).

In the next stage, the experimental data obtained for Eu (III) and Nd (III) adsorption onto functionalized cellulose were modeled using pseudo-second-order model. Linear plots of *t*/*q*_*t*_ against *t* are depicted in Fig. 6.

Table 2 contains the parameters obtained from Lagergren and Ho and McKay kinetic models used to describe the adsorption processes of Eu and Nd ions from aqueous solutions on the functionalized cellulose. By comparing the results obtained from the two kinetic models, can conclude that the adsorption processes of Eu and Nd ions on thiourea functionalized cellulose are better described by pseudo-second-order kinetic model.

**Table 2:** Kinetic parameters of the adsorption at different temperatures.

Temperature (K)	Pseudo - first - order kinetic				Pseudo - second - order kinetic		
	q_e,exp_ (mg/g)	k_1_ (1/min)	q_e,calc_ (mg/g)	R^2^	k_2_ (g/mg∙min)	q_e,calc_ (mg/g)	R^2^
Eu (III)							
298	10.26	0.0317	3.60	0.4342	4.11∙10^−3^	11.57	0.9849
308	10.33	0.0336	3.66	0.4722	4.08∙10^−3^	11.60	0.9856
318	10.37	0.0347	3.64	0.4786	4.02∙10^−3^	11.70	0.9858
Nd (III)							
298	11.68	0.0552	5.48	0.6238	3.55∙10^−3^	13.86	0.9729
308	11.71	0.0557	5.17	0.6273	3.31∙10^−3^	13.66	0.9784
318	11.74	0.0589	5.27	0.6794	3.15∙10^−3^	13.58	0.9824

This conclusion is drawn based on the values of the correlation coefficient (that is closed to 1 for the pseudo–second-order equation) and the small differences between the experimental and the computed adsorption capacities (q_e,exp_ and q_e,calc_) of the used adsorbent.

**Figure 7:**
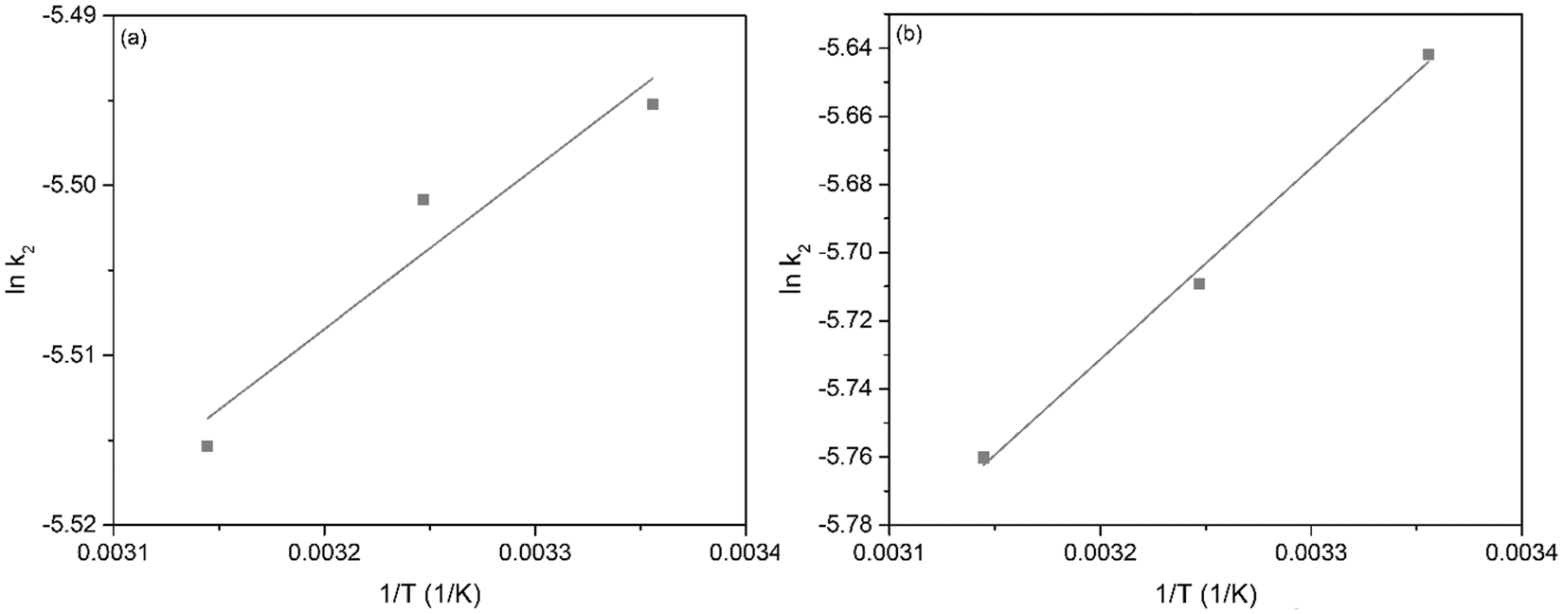
Arrhenius plot of the adsorption of (**a**) Eu (III) and (**b**) Nd (III) on the functionalized cellulose.

In Fig. 7 are depicted the Arrhenius plot obtained for the adsorption of Eu and Nd ions onto the functionalized cellulose. The activation energies associated with the studied adsorptions have been determined from the slopes of the Arrhenius linear plots. Based on the fact the Eu ions adsorption needs an activation energy of 2.99 10^−3^ kJ mol^−1^ and Nd ions adsorption needs an activation energy of 5.4 10^−4^ kJ mol^−1^, can conclude that the adsorption processes of Eu and Nd ions are physicals ones.

### Thermodynamic studies

**Figure 8:**
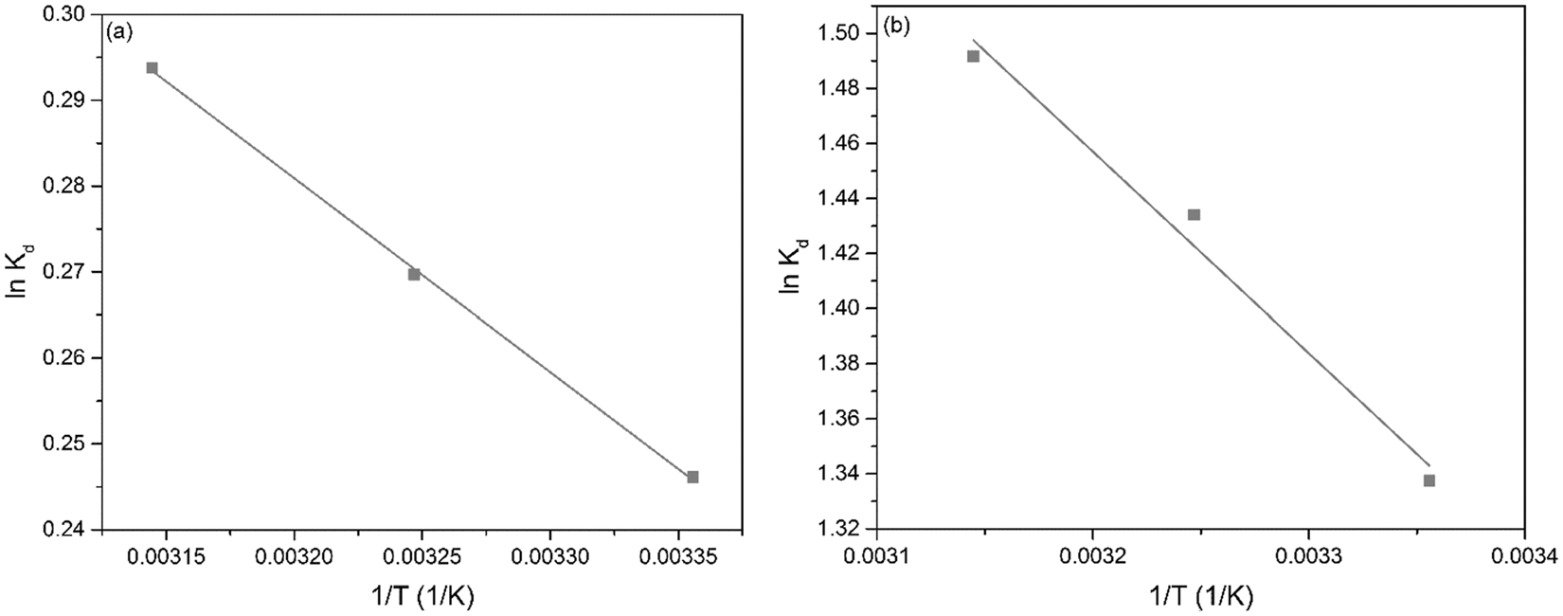
Plot of ln*K*_d_ against 1/*T* of the adsorption of (**a**) Eu (III) and (**b**) Nd (III) on the obtained material.

Figure 8 presents the linear depedence of ln*K*_d_ against 1/*T* associated with the adsorption processes of Eu and Nd ions onto the functionalized cellulose. The standard entropy change (Δ*S*°) and the standard enthalpy change (Δ*H*°) associated with studied adsorption processes are given by the slope of the line, respectively by the intersection of the lines with the Y axis. By using the computed entropy and enthalpy changes, the free Gibbs energy values for Eu and Nd adsorptions are obtained. Calculated values of the thermodynamic parameters are summarized in Table 3.

**Table 3:** The thermodynamic parameters.

	Δ*H*° (kJ/mol)	Δ*S*° (J/mol∙K)	Δ*G*° (kJ/mol)			*R* ^*2*^
			298.15 K	308.15 K	318.15 K	
Eu (III)	1.87	8.33	−0.608	−0.692	−0.775	0.9986
Nd (III)	6.08	31.52	−3.32	−3.64	−3.95	0.9682

Analyzing these parameters, one can conclude that the adsorptions on Eu and Nd ions on the modified cellulose are spontaneous processes as the free Gibbs energy has negative values in both cases. Since the standard enthalpy change has positive values, the adsorptions of Eu and Nd are endothermic processes. Positive values of standard entropy change suggest an increased disorder of the system. Since Δ*H*° had values under 80 kJ mol^−1^, can conclude that studied adsorption processes onto modified cellulose are physical adsorptions.

### Equilibrium studies

**Figure 9:**
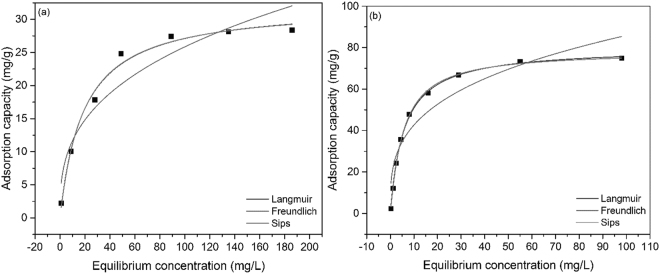
Adsorption isotherm of (**a**) Eu (III) and (**b**) Nd (III) on the functionalized cellulose with thiourea.

Adsorption isotherm of Eu and Nd ions onto thiourea functionalized cellulose are depicted in Fig. 9; based on presented data can observe that the increase of initial concentrations of the metallic ions leads to the augmentation of the adsorption capacity until a maximum is reached.

For Eu ions, the equilibrium stage was reached when the initial concentration of the metallic ions was 90 mg L^−1^. Further increasing of the initial concentration leads to a slow augmentation of the adsorption capacity until a maximum value of 27 mg g^−1^ was reached. For Nd ions, the equilibrium is reached when the initial concentration has a value of 60 mg L^−1^. Further increasing of the initial concentration leads to a slow augmentation of the adsorption capacity until reaching a maximum value of 73 mg g^−1^.

Comparing these values, we can notice a higher equilibrium concentration and a lower adsorption capacity of Eu ions and on the other hand, a lower equilibrium concentration and a higher adsorption capacity of Nd ions. This means that Nd ions are better adsorbed by thiourea functionalized cellulose.

Table 4 contains the parameters obtained by fitting the experimental data with the used isotherm models. The Freundlich isotherm model has the smaller correlation factor for both studied adsorptions, meaning that the other models have better described the adsorption of Eu and Nd on thiourea functionalized cellulose.

**Table 4:** Parameters of the Langmuir, Freundlich and Sips isotherm model.

	Langmuir isotherm				
	*q*_m,exp_ (mg/g)	*K*_L_ (L/mg)	*q*_L_ (mg/g)		*R* ^2^
Eu (III)	32	0.052	32.27		0.9914
Nd (III)	73	0.17	80.07		0.9981
	**Freundlich isotherm**				
	***K***_**F**_ **(mg/g)**	**1/** ***n***_**F**_		***R*** ^**2**^	
Eu (III)	5.24	0.34		0.9190	
Nd (III)	21.17	0.30		0.8825	
	**Sips isotherm**				
	***K***_**s**_	***q***_**s**_ **(mg/g)**	**1/** ***n***_**s**_	***R*** ^**2**^	
Eu (III)	0.05	32.09	1.01	0.9897	
Nd (III)	0.16	78.30	1.07	0.9985	

The value of the parameter 1/*n*_F_ less than 1 indicates that the adsorption process is a favorable one, described by a convex isotherm. In this case, the adsorption sites characterized by highest binding energy are occupied first, followed by the sites with lower binding energy. A value of the parameter 1/*n*_s_ approximately 1 suggests that the functionalized material have a low heterogeneity; as consequence, the mathematic equation associated to Sips’ isotherm will be the same as that of the Langmuir isotherm. As consequence, the correlation coefficients of the Langmuir and Sips isotherms are almost equals. In both cases, the computed adsorption capacities of Eu and Nd ions are close to the experimental values.

### Mathematical models

**Figure 10:**
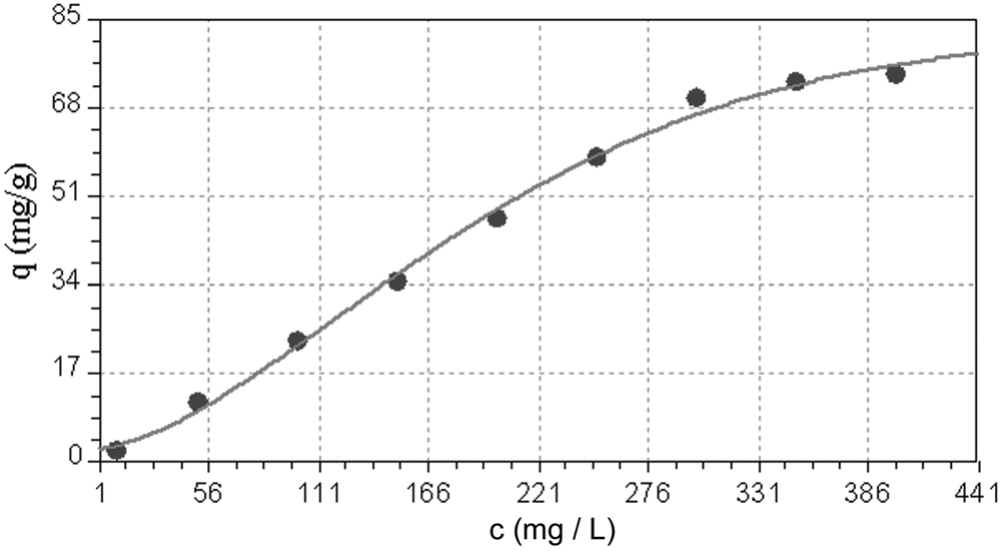
Dependence of adsorption capacity function of initial concentration.

The first model determined was that of the evolution of adsorption capacity (q) function of metallic ions concentration (c) at 298 K. This model is of Weibull type (Fig. 10), with the equation:

10 $$q=82.719-79.814\,{e}^{-0.000189\times {c}^{1.588}}$$

The standard error of the model is 2.135 and the correlation coefficient 0.998.

It can be seen that adsorption capacity q is stabilizing at an initial metallic ions concentration of about 300 mg.

The second model is that of the dependence of the adsorption capacity function of the adsorption time (t) and temperature (T) (Fig. 10):

11 $$q=0.1714\,t+0.1177\,T.$$

To test the significance of the model’s coefficients and of the model in its whole, the t-test and the F-test have been performed at the significance level of 0.05.

The values of the t-statistics corresponding to the coefficients are respectively 4.932 and 3.874, and the corresponding p-values 0.000 and 0.0043. Since the p-values are less than 0.05, the coefficients are significant. The value of the F-statistics is 174.79 and the p-value = 0.000, proving that the model is significant.

The determination coefficient is R^2^ = 0.972, showing that 97.2% of the variation of *q* is explained by the variation of *T* and *t*.

To validate the model, the residual analysis has been performed as well, at the same confidence level. We found that there is no autocorrelation of the residual, they are Gaussian (the value of the Anderson – Darling statistics is 0.293 and the p-value = 0.542), and homoscedastic (the Levene statistics is 0.02 and the p-value = 0.880). Therefore, the multiple linear model is correct from the viewpoint of statistics.

## Conclusions

Present investigation demonstrate that the new material obtained by functionalization of the cellulose with thiourea represent an effective adsorbent material for Eu (III) and Nd (III) cations removal from aqueous solutions. FTIR and EDX spectra show that the cellulose was successfully impregnated with thiourea. It was proven that for an efficient adsorption of Eu and Nd ions, the optimum adsorption parameters are: adsorbent dosage – 0.1 g of functionalized material, contact time – 30 minutes, and temperature – 298 K. The equilibrium study proved that the adsorption of rare earth elements is well described by Langmuir isotherm, the maximum adsorption capacities being of 27 mg g^−1^ for Eu (III) and 73 mg g^−1^ for Nd (III). Kinetic behavior of Eu and Nd adsorption, are better described by the pseudo–second–order models rather than pseudo–first–order model, confirming that the studied adsorptions are physical one.

The mathematical models can be used for the interpolation of the experimental values of adsorption capacity function of metallic cations initial concentrations and also function time and temperature. Mathematical model proved that the maximum adsorption capacity is reached when the initial concentration was around 300 mg/L.
